# Ion-specific ice recrystallization provides a facile approach for the fabrication of porous materials

**DOI:** 10.1038/ncomms15154

**Published:** 2017-05-02

**Authors:** Shuwang Wu, Chongqin Zhu, Zhiyuan He, Han Xue, Qingrui Fan, Yanlin Song, Joseph S. Francisco, Xiao Cheng Zeng, Jianjun Wang

**Affiliations:** 1 Key Laboratory of Green Printing, Institute of Chemistry, Chinese Academy of Sciences, Beijing 100190, China; 2 School of Chemistry and Chemical Engineering, University of Chinese Academy of Sciences, Beijing 100049, China; 3 Department of Chemistry, University of Nebraska-Lincoln, Lincoln, Nebraska 68588, USA; 4 Beijing Advanced Innovation Center for Soft Matter Science and Engineering, Beijing University of Chemical Technology, Beijing 100029, China

## Abstract

Ice recrystallization is of great importance to both fundamental research and practical applications, however understanding and controlling ice recrystallization processes remains challenging. Here, we report the discovery of an ion-specific effect on ice recrystallization. By simply changing the initial type and concentration of ions in an aqueous solution, the size of ice grains after recrystallization can be tuned from 27.4±4.1 to 277.5±30.9 μm. Molecular dynamics simulations show that the ability of the ion to be incorporated into the ice phase plays a key role in the ultimate size of the ice grains after recrystallization. Moreover, by using recrystallized ice crystals as templates, 2D and 3D porous networks with tuneable pore sizes could be prepared from various materials, for example, NaBr, collagen, quantum dots, silver and polystyrene colloids. These porous materials are suitable for a wide range of applications, for example, in organic electronics, catalysis and bioengineering.

## Abstract (web summary)

Ice recrystallization is important for a range of research fields, both applied and fundamental, however the effect of ions on this process is not well known. Here the authors discover an ion-specific Hofmeister type effect on ice recrystallization, using this phenomenon to prepare porous materials with tuneable pore sizes.

Ice recrystallization is a ubiquitous phenomenon in nature^1^,^2^,^3^,^4^, with consequences for our daily lives, including the survival of living organisms^5^,^6^,^7^,^8^,^9^ in subzero environments, food storage^10^, glacial flow in cold climates (such as Antarctica and Greenland)^11^, sea current changes^12^ and decreases in ozone concentration^13^ in the stratosphere. Ice recrystallization occurs mainly through the process of Ostwald ripening^14^. In this process, large ice crystals grow at the expense of small ones, which results in an increase in the mean crystal size and a decrease in the total number of crystals. Impurities are often included during ice recrystallization^15^,^16^,^17^. For example, it has been suggested that recrystallization of sea ice led to the formation of prebiotic materials, which may have important implications for the emergence of life^18^. Therefore, studying the effects of impurities on ice recrystallization is highly desirable^19^,^20^, particularly ion-specific effects on ice recrystallization. For example, does the ability of ice crystals to incorporate ions vary for different types of ions? Can ions significantly influence the size of ice grains after recrystallization?

Ion specificity, also known as the Hofmeister effect, was first discovered by Franz Hofmeister in 1888 (ref. 21). This effect refers to the intriguing phenomenon wherein different types of ions can have profoundly different effects on the stability of proteins. Subsequently, additional evidence for ion-specific effects on other physico-chemical properties and processes have been revealed, such as effects on surface tension, protein folding, protein crystallization, enzymatic activity and colloidal assembly^22^,^23^,^24^,^25^,^26^,^27^. Most recently, an ion-specific effect on heterogeneous ice nucleation has been reported^28^. However, to our knowledge, the effect of ion specificity on ice recrystallization has not been studied.

Herein, we report both experimental and theoretical evidence of ion specificity in regulating ice recrystallization. We find that the size of ice grains after recrystallization can be regulated by more than one order of magnitude by simply changing the type or concentration of ions in the aqueous solution at the initial stage. This phenomenon can be understood on the molecular level through molecular dynamics (MD) simulations. Specifically, our simulation demonstrates that the ability of ice crystals to incorporate ions and the stability of the liquid layer between crystal domains both exhibit an ion-specific effect. More remarkably, we show that ion-specific recrystallized ice crystals can be exploited as a template-based approach for the facile fabrication of various 2D and 3D porous materials, and the pore size can be controlled simply by regulating the ice grain size via changes in the type and concentration of ions. As an alternative to conventional ice templates created via a directional ice growth process, this method provides a different approach to fabricating porous materials^29^,^30^,^31^,^32^,^33^.

## Results

### Effect of ions on ice recrystallization

**Figure 1:**
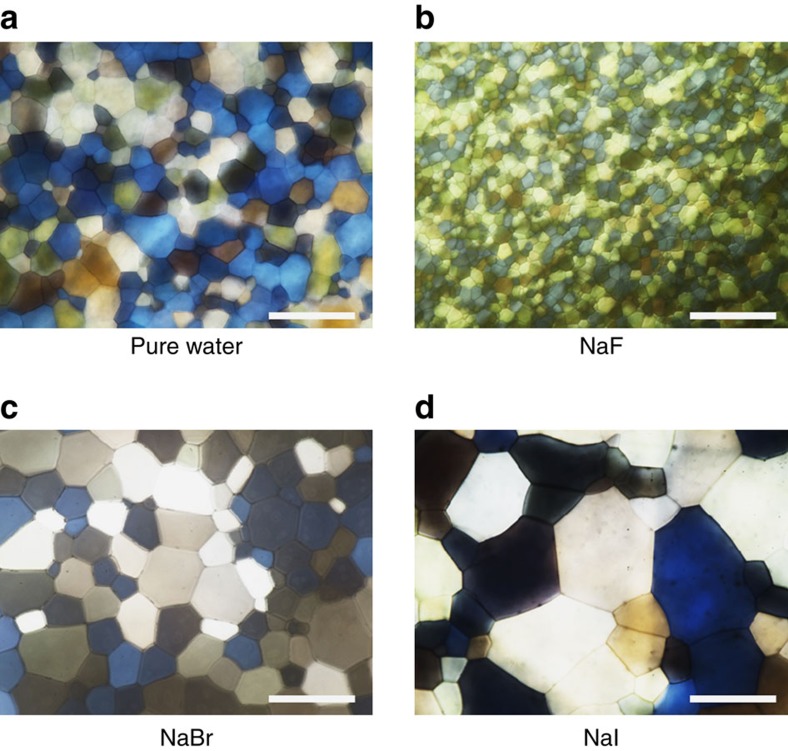
Polarized optical microscopy images of polycrystalline ice crystals. (**a**–**d**) show the typical grain size of ice crystals after rapid freezing and annealing at a higher temperature of pure water, NaF, NaBr and NaI aqueous solutions, respectively. Clearly, anions have a distinct effect on ice recrystallization. Aqueous solution droplets (20 μl) were dropped onto a silicon wafer substrate from a height of 1.5 m. The wafer surface temperature was −60 °C, and the substrate was placed in a N_2_ environment to prevent water condensation and ice formation. Then, the ice crystals were annealed at −6 °C for 45 min. The concentration of the salt solution was 0.01 M. The scale bar is 200 μm.

We quantitatively evaluated the ability of various ions to regulate ice recrystallization with the ‘splat-cooling' assay^34^ (Supplementary Fig. 1). Briefly, aqueous solutions of different ions were rapidly frozen (at quenching temperatures ranging from −30 to −90 °C) on a flat solid substrate to form polycrystalline ice crystals, and then the ice crystals were annealed at a higher temperature (for example, −6 °C). During the annealing process, the mean size of the ice grains increases while the total number of ice grains decreases (Supplementary Fig. 2). Figure 1 shows typical annealed ice grains obtained from pure water and three types of salt solutions (0.01 M NaF, NaBr and NaI). The mean grain size of the ice crystals obtained from pure water is 158.3±11.2 μm (Fig. 1a) and is much larger than that obtained from the NaF solution (34.2±4.7 μm; Fig. 1b). In stark contrast, the mean grain size of ice crystals obtained from the NaI solution is 228.7±20.1 μm and is much larger than that obtained from pure water. Interestingly, the mean grain size of ice crystals obtained from the NaBr solution (165.8±12.5 μm) is comparable to that obtained from pure water. Clearly, the mean size of ice crystals can be regulated to be either much smaller than, comparable to or much larger than that obtained from pure water, simply by changing the anions in the initial solution.

**Figure 2:**
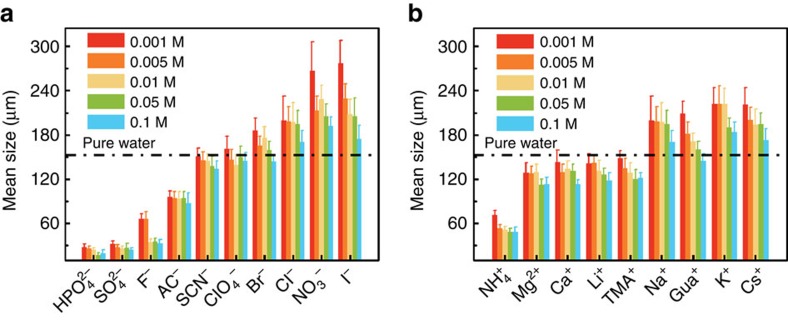
Effect of ions on tuning ice recrystallization. (**a**) The effect of anions in tuning the ice crystal size. Na^+^ was used as the common counter-ion. (**b**) The effect of cations in tuning the ice crystal size. Cl^−^ was used as the common counter ion. Five concentrations were tested for each ion. The mean grain sizes obtained from aqueous solutions of different ions were measured after the polycrystalline ice was annealed at −6 °C for 45 min, while the initial quench temperature was −60 °C. Error bars represent the s.d. of the size of thirty ice crystals. The dash-dot line is the mean grain size from pure water as a reference.

To confirm the ion-specific effect on ice recrystallization, several more ions were studied (Fig. 2). The mean grain size of ice crystals obtained from salt solutions with various anions but the same Na^+^ cation follows the sequence: 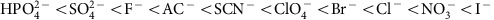. Note that the ice grains produced from the 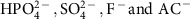 salt solutions are much smaller than those obtained from pure water, whereas the ice grains produced from 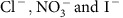 are much larger than those obtained from pure water. The ice crystal grain size from salt solutions of different cations but with the same Cl^−^ anion follows the sequence 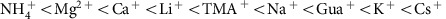 (Fig. 2b). As shown in Fig. 2b, the ice crystal grains obtained from 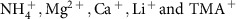 solutions are smaller than those obtained from pure water, and the ice crystal grains obtained from Na^+^, Gua^+^, K^+^ and Cs^+^ solutions are larger than those obtained from pure water. Note that the ability of anions to modify ice recrystallization is greater than that of cations, which is consistent with other ion specificities^35^. Therefore, our work demonstrates evidence of the Hofmeister effect on ice recrystallization.

### Various factors that can affect ice recrystallization

**Figure 3:**
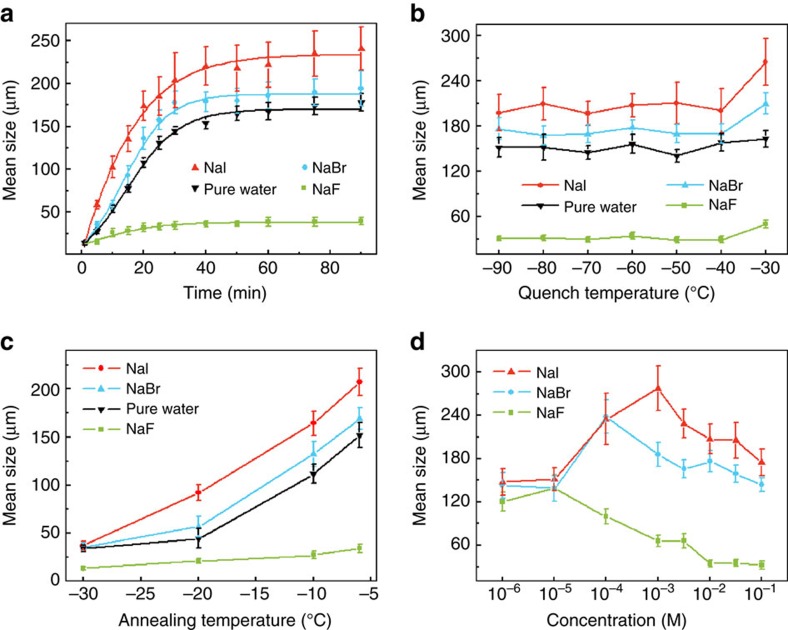
Grain size variation. The mean grain size of the ice crystals recrystallized from pure water and NaF, NaBr and NaI aqueous solutions at: (**a**) Different annealing times with the quench temperature of −60 °C and annealing temperature of −6 °C; (**b**) Various quench temperatures with an annealing temperature of −6 °C and annealing time of 45 min; (**c**) Different annealing temperatures with a quench temperature of −60 °C and an annealing time of 45 min; (**d**) Various concentrations of salts with quench and annealing temperatures of −60 °C and −6 °C, respectively, and an annealing time of 45 min. Note that the mean size of ice crystals obtained in NaF, NaBr and NaI peaked at concentrations of 10^−5^ M, 10^−4^ M and 10^−3^ M, respectively. For the experiments shown in (**a**–**c**) the concentration of salts was 0.01 M. The error bars represent the s.d.

To gain insight into the ice recrystallization process, the size evolution of the ice crystals as a function of the annealing time was analysed (Supplementary Figs 3 and 4), as shown in Fig. 3a. For the ice crystals obtained from three aqueous solutions and from pure water, all the ice grains experience an initial growth at the expense of small ice crystals and then level off after ∼45 min. Note that both the initial growth rate and the mean grain size after 45 min increase in the sequence of NaF<pure water<NaBr<NaI (Fig. 3a).

There are two ways to influence the ice recrystallization process with ions: through nucleation and through ice growth^34^,^36^. To determine which process is more important, the effects of the quench and annealing temperatures on the mean size of the ice grains from different salt solutions (0.01 M) and pure water were investigated, as shown in Fig. 3b,c. Our experiments show that the quenching temperature only slightly affects the mean grain size, which implies that the nucleation process plays a small role in the mean grain size at this ion concentration, and the nucleation process is mainly dictated by the quenching temperature. By contrast, the annealing temperature plays a key role in dictating the mean size at this ion concentration, which indicates a pronounced effect of the crystal growth process in ice recrystallization (Supplementary Tables 1–5).

We further studied the effect of the salt concentration on the ion specificity in regulating ice recrystallization (Fig. 3d; Supplementary Fig. 5). When the salt concentration increases stepwise from 10^−6^ to 10^−1^ M, the mean grain size of recrystallized ice crystals follows the sequence of F^−^<Br^−^<I^−^. Moreover, the grain size first increases and then decreases for all ice crystals obtained from NaF, NaBr and NaI solutions. The mean grain size of ice crystals from NaF, NaBr and NaI solutions peaks at salt concentrations of 10^−5^ M, 10^−4^ M and 10^−3^ M, respectively, and the corresponding values of the grain size are 138.9±18.2 μm, 238.2±22.6 μm and 277.5±30.9 μm. The same sequence is observed in the effects of the quench temperature and the annealing time and temperature (Supplementary Fig. 3a–c, respectively). Note that previous studies of the evolution of the freezing potential^37^,^38^ as ice crystals grow in aqueous salt solutions have revealed the same trend as that of the effect of the salt concentration on ice recrystallization. Namely, both exhibit a trend wherein the effect is to first increase and then decrease, which suggests that the regulation of ice recrystallization by ions in our experiments may be due to different amounts of ions in the ice crystals.

### Mechanistic investigation of ice recrystallization

Questions arise as to (1) why all the ions lead to this generic trend, namely, a maximum in the mean size versus ion concentration curve (Fig. 3d) and (2) why the critical ionic concentration (corresponding to the maximum in Fig. 3d) follows the sequence of F^−^<Br^−^<I^−^? Previous studies have indicated that the ions generally suppress ice nucleation^39^. In other words, due to the added ions, the likelihood of nucleus formation in aqueous solutions tends to decrease in comparison to that in pure water. Moreover, at extremely low-salt concentrations, the ions affect ice nucleation more strongly than ice growth. As a result, the mean grain size of ice crystals should increase if ice formation occurs at very low-salt concentrations, regardless of the type of ions. Note that this conclusion is consistent with a previous study of the ion-specific effect on heterogeneous ice nucleation^28^. In that study, the experiments showed that the ion-specific effect on ice nucleation followed the sequence F^−^>Br^−^>I^−^. In other words, among the three anions, F^−^ leads to the largest decrease in the incipient temperature of ice formation. As a result, the critical ionic concentration is expected to follow the sequence F^−^<Br^−^<I^−^, because the lowest concentration of F^−^ is required to suppress ice nucleation. By contrast, at a relatively high-salt concentration, ice growth is expected to be seriously affected regardless of the type of ion. Therefore, an increase in the ion concentration should lead to a decrease in the grain size.

**Figure 4:**
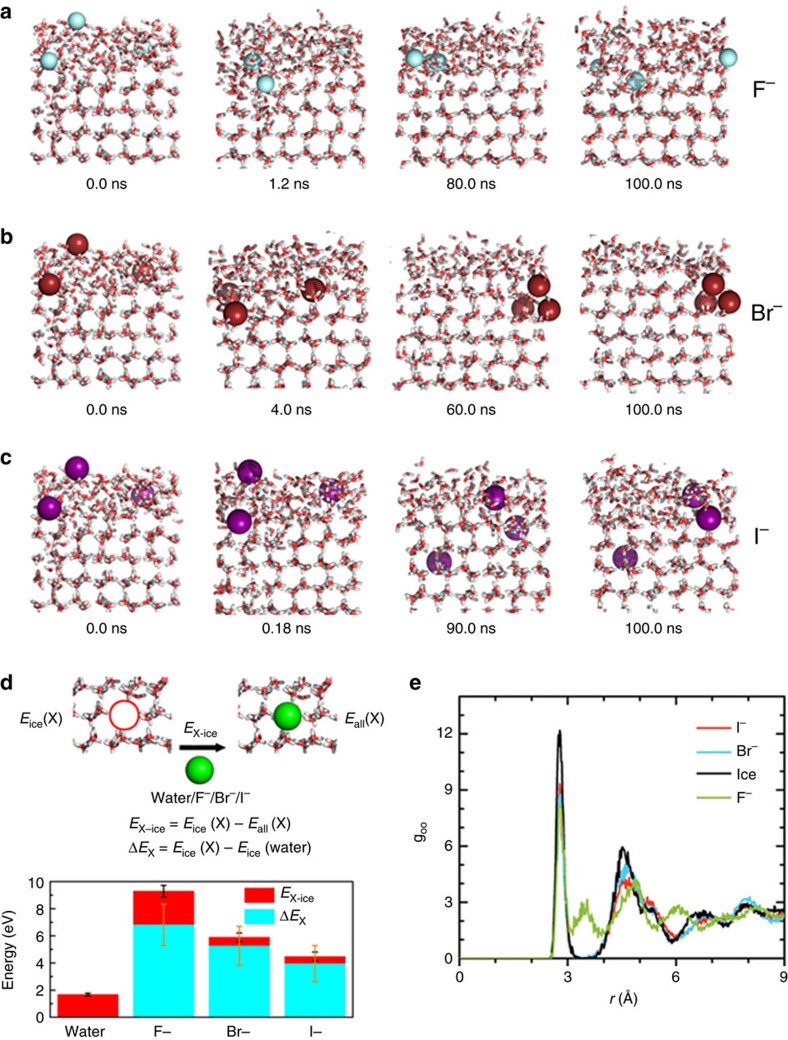
Molecular dynamics simulations. Snapshots of the MD simulations for illustrating the effect of (**a**) F^−^, (**b**) Br^−^ or (**c**) I^−^ ions on ice formation at −13 °C. (**d**) Results of test MD simulation (see Methods) for computing average interaction energy (*E*_X-ice_) of a water molecule or an ion with the surrounding water molecules in the bulk ice are depicted by the height of red bars (results being averaged over 50 configurations in the last 100 ps of the 1 ns test MD simulations). Here, *E*_all_(X) denotes the total potential energy of the system after the replacement of two tagged water molecules with X (X=water, F^−^, Br^−^ or I^−^) and Na^+^. The potential energy increase (Δ*E*_X_) for the pure ice crystal due to the presence of the embedded ions is marked by the height of cyan bars. The root-mean-square error bars (black and yellow) are calculated based on the data of 50 configures in the last 100 ps. (**e**) Computed radial distribution functions of O atoms in pure ice and O atoms surrounding the halide ions.

To gain more insight into the mechanism underlying the ion-specific effect on ice recrystallization, classical MD simulations were performed (see Methods section for details). Snapshots of the systems with F^−^(Supplementary Fig. 6), Br^−^ and I^−^ anions at different simulation times are shown in Fig. 4a–c, respectively. The results clearly indicate that F^−^, Br^−^ and I^−^ anions can be incorporated into the ice crystal. As shown in Fig. 4a and Supplementary Movie 1, the addition of ions induced vicinal ice melting at an early stage (1.2 ns), whereas at a later equilibrium stage (100 ns), all F^−^ ions remain in the liquid water phase (with 154±21 water molecules). This result is in stark contrast to the situation for Br^−^ and I^−^ ions, as shown in Fig. 4b,c and Supplementary Movies 2 and 3. Only one ion was left in the liquid water phase (with 114±12 water molecules for Br^−^ and 87±19 water molecules for I^−^ in liquid phase, respectively) and the other two ions were dissolved in the ice phase at the later equilibrium stage (100 ns). Similar phenomena are also observed at the lower temperature (Supplementary Fig. 7). All these results indicate that (1) F^−^ is the least likely to be incorporated into the ice phase; and (2) the thickness of the remaining vicinal liquid water layer follows the trend F^−^>Br^−^>I^−^. Both conclusions provide an explanation as to why the mean grain size after equilibration follows the sequence F^−^<Br^−^<I^−^. Because the F^−^ ions tend to remain in the vicinal liquid water layer during ice growth, further ice crystal growth is blocked, which yields smaller ice grains than grains obtained with Br^−^ or I^−^ ions. Note that the same qualitative conclusions can be drawn from independent test simulations with the conventional Nosé–Hoover thermostat (Supplementary Fig. 8).

To quantitatively evaluate the tendency of halide ions to be incorporated into an ice crystal, we performed another series of test MD simulations to compute the average interaction energy of an ion with the surrounding water molecules in the bulk ice (*E*_X-ice_) (calculation values are marked by the height of red bars in Fig. 4d). For comparison, the interaction energy of a water molecule in bulk ice is also computed. Our simulation shows that this energy is ∼1.69±0.10 eV, which is approximately four times that of the typical hydrogen-bonding energy in bulk ice. However, the interaction energy of an ion with the surrounding water molecules in the bulk ice is much greater, and their magnitudes follow the sequence F^−^>Br^−^>I^−^. The largest difference in the required energy between F^−^ and a water molecule in the bulk ice indicates the highest degrees of disruption to the local ice structure near the F^−^ ions, or the lowest tendency of F^−^ to be incorporated into the ice crystal. We further quantified the degrees of disruption to the local ice structure due to the ions by computing the radial distribution functions (RDF) of O atoms in pure bulk ice and the O atoms surrounding the halide ions (Fig. 4e). The first peak appears at a distance of 2.78 Å, regardless of the presence or absence of ions. The second peak at a distance of ∼3.46 Å, however, is entirely due to the presence of F^−^, whereas the second peak at a distance of ∼4.51 Å is observed for the pure ice or in the presence of Br^−^ and I^−^. These results offer a molecular-level explanation for why F^−^ is the least likely ion to be incorporated into the ice crystals. This conclusion can be further supported by the potential energy increase in the pure ice crystal due to the presence of the embedded ions (the values of energy increase are marked by the height of cyan bars in Fig. 4d). With the addition of ions, the net potential energy of the bulk ice (excluding the central water molecule or the ion) is increased, and this energy increase follows the sequence F^−^>Br^−^>I^−^, which indicates that the tendency of the halide ions to be incorporated into the ice slab follows the sequence F^−^<Br^−^<I^−^. Therefore, a molecular-level mechanism of ion-specificity in ice recrystallization is provided.

### Facile synthesis of 2D and 3D porous materials

**Figure 5:**
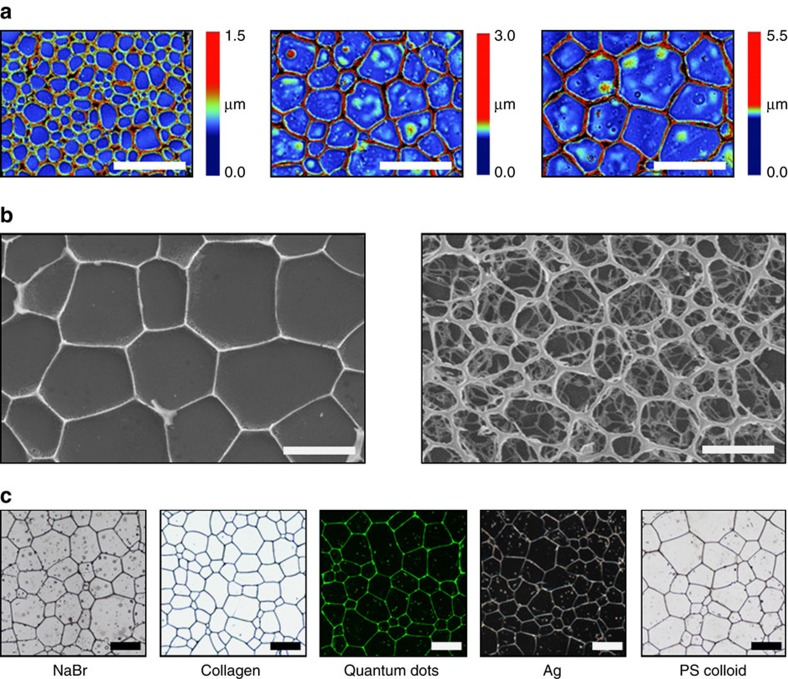
Porous materials prepared using recrystallized ice crystals as template. The images in (**a**) show that the size of the 2D meshes can be easily regulated by changing the ions or the ion concentration in the initial aqueous solution. (**b**) The 2D and 3D porous materials can be prepared with recrystallized ice crystals as the template. (**c**) Various materials such inorganic salts, collagen, quantum dots, metallic nanoparticles and polystyrene colloids can be used to prepare porous materials. All the scale bars are 200 μm.

As shown above, the size of recrystallized ice grains can be easily regulated by changing the ions or ion concentration in the initial aqueous solution, and the variation in the size of the ice grains can be more than one order of magnitude. Another important feature of recrystallized ice crystals is that most of the impurities are excluded from the ice grain and left at the grain boundaries after recrystallization^40^,^41^. Therefore, we can take advantage of these features and utilize the recrystallized ice grains as templates to fabricate various 2D and 3D porous materials with different pore sizes. Figure 5a shows a series of 2D collagen meshes with variable pore sizes, which demonstrates that the pore size can be tuned by either changing the type or concentration of ions, whereas Fig. 5b displays that 2D and 3D porous materials can be fabricated (Fig. 5b) in view of their potential applications^42^,^43^. The porous materials^44^,^45^,^46^ can be fabricated by first recrystallizing aqueous salt solutions with various materials, such as inorganic materials, biopolymers, quantum dots, metallic particles and polymer colloids (Supplementary Figs 9–14), and then dried under vacuum. As exhibited in Fig. 5c, the same general method can be used to prepare porous materials from a wide range of constituents and for various applications. For example, pore size plays a key role in bioengineering applications, such as cell attachment^47^, cell culturing^48^,^49^ and cell separation.

## Discussion

In summary, we have shown clear evidence that ions can significantly affect ice recrystallization, but the degree of influence differs dramatically, depending on the type of ion. Remarkably, we find that these ions could be ranked as a Hofmeister series based on their ability to tune the ice recrystallization. MD simulation results indicate that, during the ice recrystallization, relatively high charge density ions such as F^−^ prefer to stay at the ice-water interface and thus inhibit ice growth. By contrast, ions of relatively low charge density, such as I^−^, are more likely to be incorporated into ice crystals and thus inhibit ice growth to a lesser extent. Most importantly, we found that when the ice crystals were used as templates for fabricating 2D and 3D porous materials, the pore size can be easily tuned by regulating the mean grain size with different ions or ion concentrations. The generic approach of using recrystallization to regulate ice templates provides a way to fabricate 2D and 3D structures based on a wide variety of materials, from small molecules to nanoparticles.

## Methods

### Ice recrystallization assay

All salts were purchased from Sigma-Aldrich. And the water used for preparing the electrolytes solutions was Milli-Q (18.2 MΩ) water. Ice recrystallization was performed via the splat-cooling method as described previously^34^,^50^ (also see Supplementary Methods). The experimental apparatus was composed of a Nikon polarized optical microscope (LV100ND, Japan) equipped with a digital camera (Nikon Y-TV55, Japan) and a Linkman (C194) cooling stage shown in Supplementary Fig. 1. The experimental procedure is described as follows: a 20 μl droplet of electrolyte solution was dropped onto the cover glass precooled to −60 °C from a height of 1.5 m forming a piece of polycrystalline ice. The temperature was increased to −6 °C at a rate of 25 °C min^−1^, and then the samples were annealed at this temperature for 45 min. Thereafter, the ice was imaged randomly. For every sample, five experimental runs were performed. To plot each figure, the size of the largest 10 grains was measured using Nano-Measure 1.2. Among these 50 data, 30 corresponding to the largest grains were chosen to calculate the mean size. One way ANOVA and 2^2^ factorial design was used to analyse the mean size results (Supplementary Tables 1–6).

### Synthesis of 2D and 3D porous materials

Collagen (from bovine Achilles tendon, hydrolysis product, 1000 Dalton) was purchased from J&K Chemicals. Aqueous salt solutions of various materials (0.01 M NaF, NaBr and NaI) were splat-frozen and annealed at −6 °C for 45 min. Then, the samples were freeze-dried using a freeze dryer (FD-1A-50) to obtain the 2D and 3D materials. To obtain the 2D porous collagen, the polycrystalline ice needs to be as thin as possible. Hence we set the splat-freezing height at 1.8 m to form a polycrystalline ice layer <10 μm (ref. 51). After annealing at −6 °C for 45 min, only one layer of polycrystalline ice is formed. When preparing 3D structures, the splat-freezing height was set at 1 m, and after one droplet was splat-frozen, another droplet was splat-frozen on the top of the previous one. The splat-freezing process was repeated many times as needed to form multi-layered polycrystalline ice. Then multi-layered polycrystalline ice was annealed at −6 °C for 45 min. After sublimation of ice crystals, the 3D interconnected network was retained. The 1 wt% collagen solution was utilized to obtain 2D one-layer meshes and 5 wt% collagen solution was used to make 3D interconnected network.

### Molecular dynamics simulations

The MD simulation system consisted of 1280 water molecules, 3 Na^+^ ions and 3 anions (F^−^, Br^−^ or I^−^). The initial configuration was prepared by bringing together an ice slab of 958 water molecules (per supercell) with two separate liquid slabs, each having 164 water molecules (per supercell). All three slabs were separately equilibrated at −13 °C for 1 ns. Next, the two liquid slabs were placed in contact with the left and right surfaces of the ice slab (the surface normal is in the *z*-direction) (Supplementary Fig. 6), within a larger simulation box with a length of 10 nm in the *z*-axis and a lateral area of 2.72 × 2.35 nm^2^. The system was then equilibrated for 1 ns. Last, three water molecules in the left liquid water slab were replaced with Br^−^ anions, and three water molecules in the right liquid water slab were replaced with Na^+^ cations. The entire system was then equilibrated for ∼1 ns. Thus, the whole system was effectively a quasi-two-dimensional hybrid slab. Periodic boundary conditions were applied in the *x* and *y* directions.

The MD simulations for the two-phase systems illustrated above were carried out using the LAMMPS^52^ software. The TIP4P/ICE water model was adopted. The interaction parameters for ions were taken from the OPLS force field. The LINCS algorithm^53^ was employed to treat the constraints in the model. The van der Waals forces were truncated at 10 Å. When the long-range dispersion correction was applied, the relative root-mean-square error in force was found to be <10^−4^. The particle-mesh Ewald method^54^ was used to treat long-range Coulomb interactions. The particle-mesh Ewald parameters were chosen such that the relative error in force was comparable to that of the Lennard–Jones interactions (<5 × 10^−4^). Specifically, the real space cut-off distance was 10 Å. The charge grid spacing was 1 Å, and the fourth order B-splines were used for interpolation. The leap-frog Verlet integration algorithm with a time step of 1 fs was used for the MD simulation. To rule out the latent-heat-dissipation effects, the hybrid microcanonical-canonical ensemble is employed^55^. As shown in Supplementary Fig. 16, the molecules whose centres of mass lie in a half region of the simulation box (z<5 nm), away from the interface, are subjected to *NVT* dynamics (*T*=−13 °C), while the molecules lie in the other half region of the simulation box (z>5 nm) are subjected to *NVE* dynamics (the half region including the solid/liquid interface)^55^. In our *NVE/T* MD simulations, the temperature control was implemented by separated Nose-Hoover four-element chain thermostats for each molecule with all four time constants equal to 1 ps (refs 56, 57, 58, 59).

### Interaction energy calculation

In addition, independent test MD simulations (all 1 ns) were carried out to compute average interaction energy of an ion with the surrounding water molecules in the bulk ice *E*_X-ice_ (Fig. 4d,e). For these test MD simulations, the system consists of 430 water molecules (representing bulk ice phase), 1 Na^+^ and 1 anion (F^−^, Br^−^ or I^−^). The initial configuration was prepared by replacing two tagged water molecules in ice with 1 Na^+^ and 1 anion. These test MD simulations were performed at a constant pressure and temperature (*NPT*) ensemble with using the conventional Nosé–Hoover thermostat (−13 °C). The last 100 ps data (50 configurations) were collected for the averaged potential-energy calculations. Another series of test MD simulations using the same system size were performed with slightly different Lennard–Jones energy (*ɛ*) and particle-size (*σ*) parameters of the anions (Supplementary Fig. 15). The Gromacs software was utilized for these test MD simulations.

### Data availability

The data that support the findings of this study are available on request from the corresponding authors (J.W or X.C.Z.).

## Additional information

**How to cite this article:** Wu, S. *et al*. Ion-specific ice recrystallization provides a facile approach for the fabrication of porous materials. *Nat. Commun.* **8**, 15154 doi: 10.1038/ncomms15154 (2017).

**Publisher's note:** Springer Nature remains neutral with regard to jurisdictional claims in published maps and institutional affiliations.

## Supplementary Material

Supplementary Information Supplementary Figures, Supplementary Tables, Supplementary Methods and Supplementary References

Supplementary Movie 1 Molecular dynamics simulation (100 ns) for a liquid-ice interfacial system with three ions initially located in the liquid water thin film in contact with bulk ice I.

Supplementary Movie 2 Molecular dynamics simulation (100 ns) for a liquid-ice interfacial system with three ions initially located in the liquid water thin film in contact with bulk ice I.

Supplementary Movie 3 Molecular dynamics simulation (100 ns) for a liquid-ice interfacial system with three ions initially located in the liquid water thin film in contact with bulk ice I.
