# Supplementary material for: Ion-specific ice recrystallization provides a facile approach for the fabrication of porous materials
Source: Nat Commun. 2017 May 2;8:15154. doi: 10.1038/ncomms15154 (PMC5418576; doi:10.1038/ncomms15154)
Supplement: Supplementary Information — Supplementary Figures, Supplementary Tables, Supplementary Methods and Supplementary References [file ncomms15154-s1.pdf]

Supplementary Methods

Synthesis of Ag nanoparticle. $AgNO_3$ (ACS regent, 99%), Polyvinyl pyrrolidone (PVP, Mw = 55000) and glycol (EG, Vetetc™ reagent grade, 98%) were purchased from Sigma-Aldrich. Water used in all experiments was the Milli-Q (18.2MΩ) water. Silver nanoparticles were synthesized by using a modified polyol reduction method[1] with polyvinyl pyrrolidone (PVP, Mw = 55000) as the capping agent and ethylene glycol (EG) as a reducing agent. Synthesis details: 400 mg $AgNO_3$ and 800 mg PVP were added into a three-necked flask containing 40 mL EG under vigorous stirring. The mixture was heated in an oil bath and maintained at 160 ^{o}C for 15 min. After cooling to room temperature, the mixture was centrifuged to remove the excess PVP.

Ice template structure making. Salts meshes making: NaF, NaBr and NaI solutions (concentration, 0.1 M) were splat-frozen and annealed at -6 ^{o}C for 45 min, then freeze-dried with freeze dryer (FD-1A-50). Quantum dots (CdSe capped by PEO) polycrystalline ice: quantum dots were dissolved into salt solutions (NaF, NaBr and NaI, concentration, 0.01 M). Then the mixtures were splat-freezing and annealed at -6 ^{o}C for 45 min. Ag nanoparticles meshes: quantum dots were dissolved into salts solutions (NaF, NaBr and NaI, concentration, 0.01 M). Then the mixtures were splat-freezing and annealed at -6°C for 45 min. Next, the mixtures were splat-freezing and annealed at -6 ^{o}C for 45 min. The poly(styrene-methyl methacrylate acrylic acid) (PS) nanoparticles with a mean diameter of 450 nm were synthesized from our laboratory using previous synthesis method[2]. The 450 nm PS nanoparticles were assembled by using the similar freezing-annealing-drying procedure. Optical and fluorescence microscopy images were collected by using Nikon optical microscope (LV100ND, Japan) equipped with a digital camera (Nikon Y-TV55, Japan). SEM images were collected by using a JEOL-7500 scanning electron microscope

operated at 10 kV.

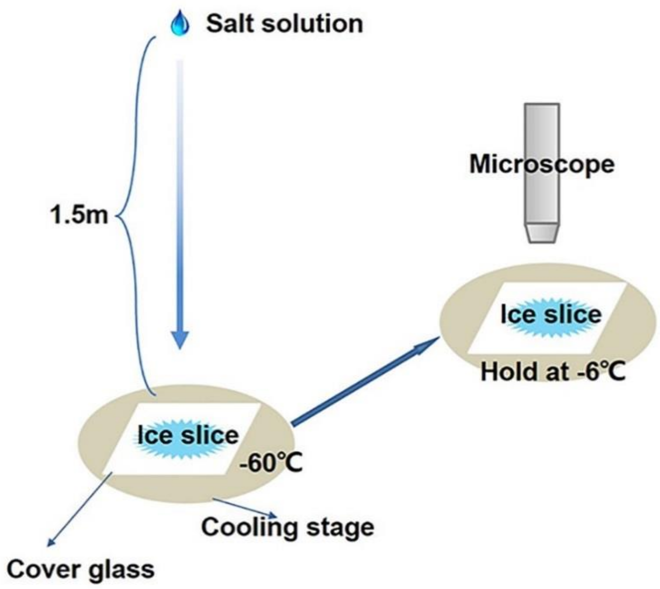

Supplementary Figure 1│The apparatus for ice recrystallization essay. The experiment apparatus is composed of a Nikon polarized optical microscope (LV100ND, Japan) equipped with a digital camera (Nikon Y-TV55, Japan) and a Linkman (C194) cooling stage.

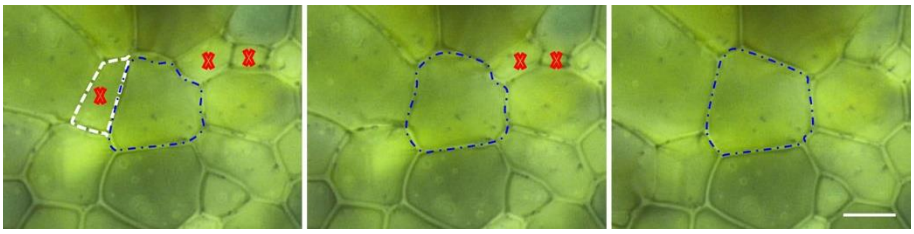

Supplementary Figure 2│The growth process of ice grains during annealing (Ostwald ripening). The large ice crystals grow at the expense of small ones. The white scale bar is 100 μm.

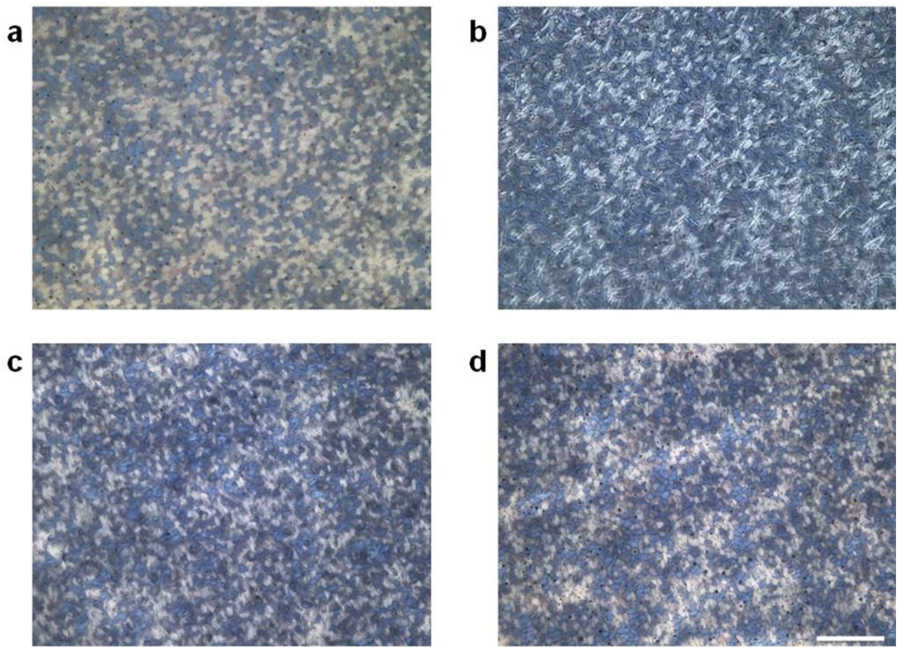

Supplementary Figure 3 | Polarized optical microscopic images of polycrystalline ice crystals from a, pure water, b, NaF, c, NaBr and d, NaI at 0 min of annealing. The concentration of salts is 0.001 M. The white scale bar is 100 μm. Topologies of the polycrystalline ice are similar, and the average size of ice grain from pure water and salts are also similar.

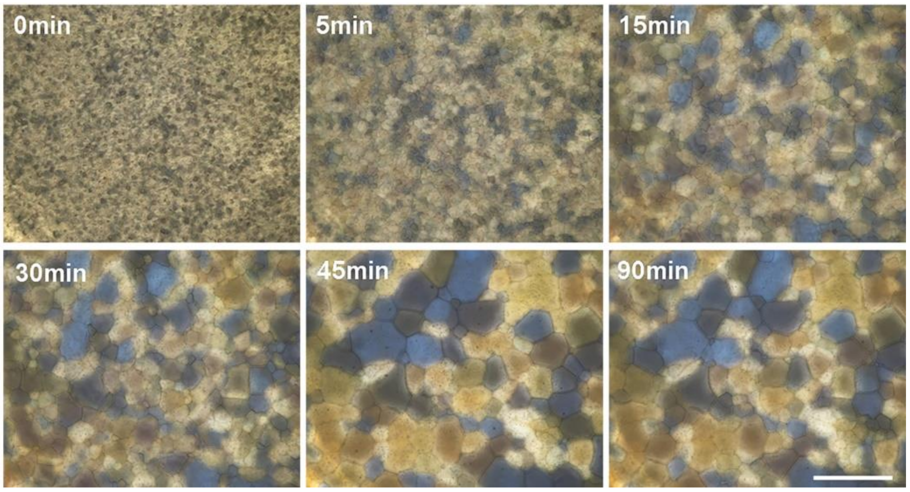

Supplementary Figure 4 | Polarized optical microscopic images of polycrystalline ice crystals from 0.01 M NaBr *versus* the annealing time. The quench temperature is -60.0 ^{o}C, and the annealing temperature is -6.0 ^{o}C. White scale bar is 400 μm.

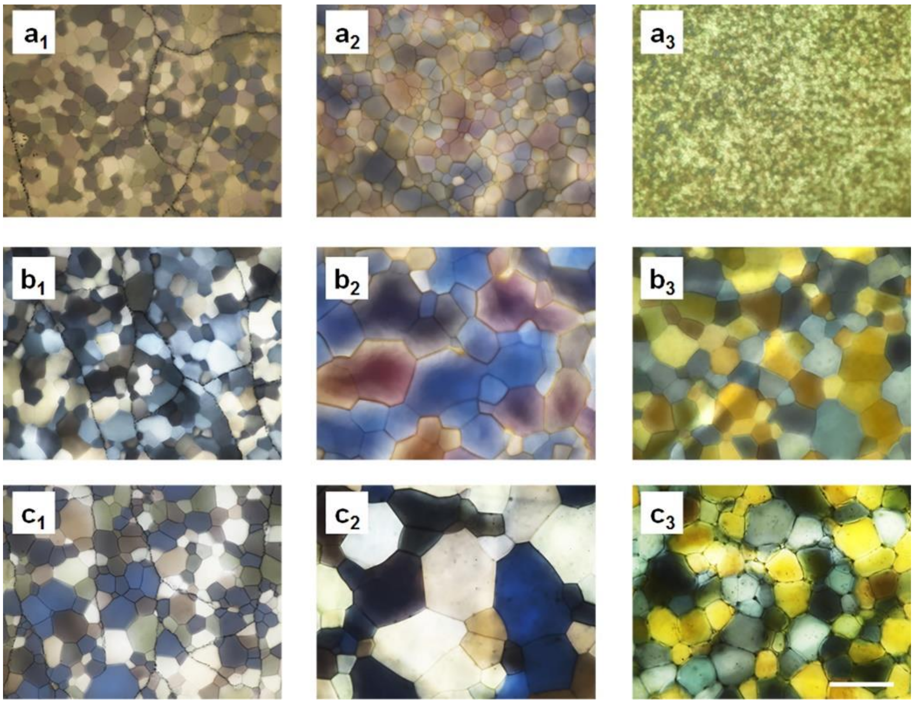

Supplementary Figure 5│Polarized optical microscopic images of polycrystalline ice crystals from a, NaF, b, NaBr and c, NaI with different concentration after annealing for 45 min at -6 ºC. Concentration, a_1, 10^{-6} M, a_2, 10^{-5} M, and $a_{3,}$ 10^{-2} M; b_1 10^{-6} M, b_2, 10^{-4} M, and b_3, 10^{-2} M; c_1 10^{-5} M, $c_{2,}$ 10^{-3} M, and c_3, 10^{-1} M. The mean grain size for ice crystals from NaF, NaBr and NaI solutions peaks at the salt concentration of 10^{-5} M, 10^{-4} M and 10^{-3} M, respectively. The corresponding values of the grain size are 138.9 ± 18.2 µm, 238.2 ± 22.6 µm and 277.5 ± 30.9 µm. The white scale bar is 200µm.

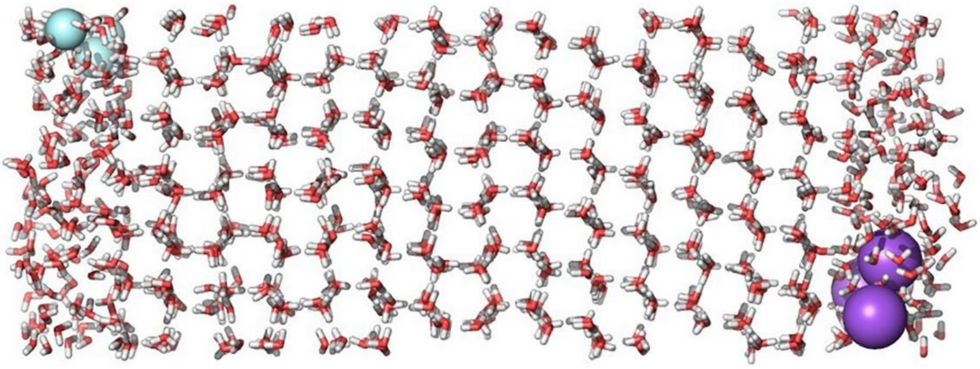

Supplementary Figure 6 | A snapshot (at 100.0 ns) of the equilibrated ice/water slab system at -13 ℃. The three F^- ions (small cyan spheres) are located in the left liquid water slab while the three Na^+ ions (big purple spheres) are located in the right liquid water slab.

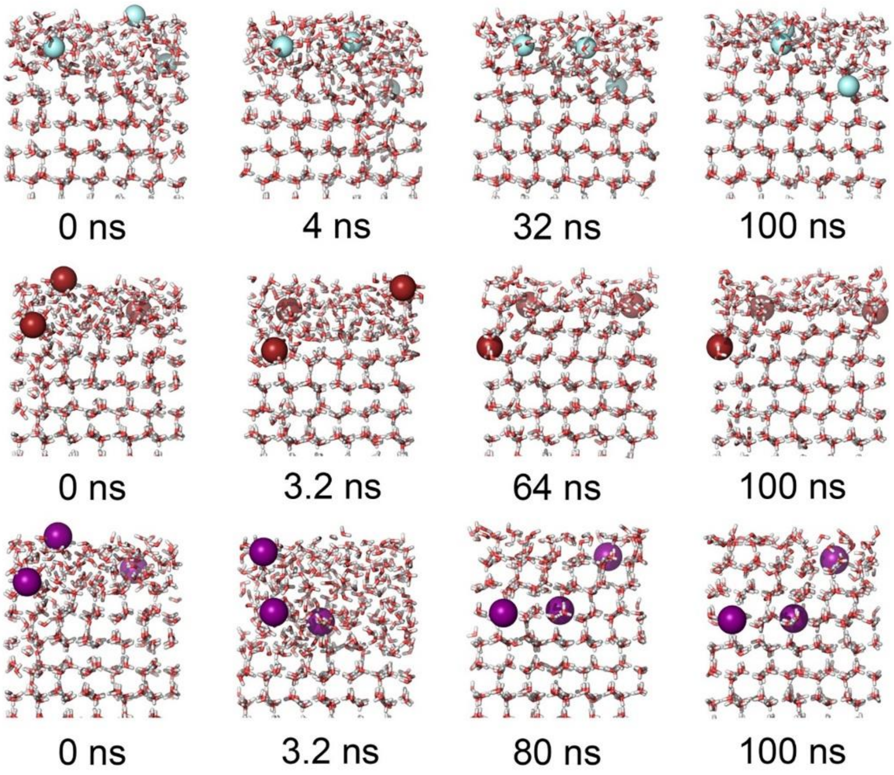

Supplementary Figure 7 | Snapshots of the MD simulation at different time for showing the effect of the addition of different anions on the ice formation: (a) F^-, (b) Br^- and (c) I^- at -43 ℃.

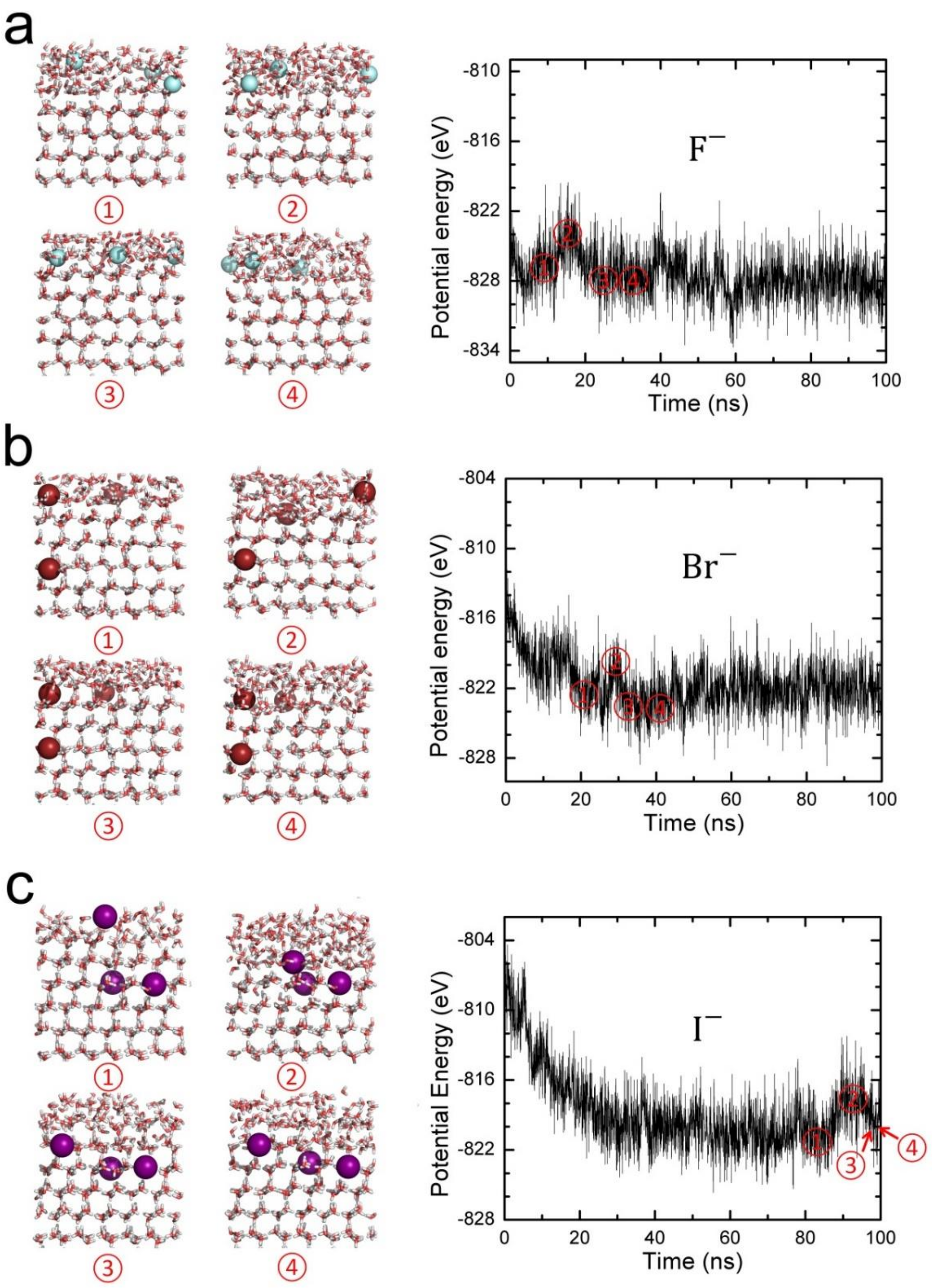

Supplementary Figure 8 | Snapshots of testing MD simulations (in $NP_{xy}T$ ensemble but with the conventional Nosé-Hoover thermostat), and the corresponding interaction energy along the MD trajectory for (a) F^-, (b) Br^- or (c) I^- ions at -23 °C. For these testing simulations with the conventional Nosé-Hoover thermostat, the Gromacs software package was used. From the figure, the energy required for incorporating an F^- from liquid water into bulk ice is about 2.8 eV, while the energy required for incorporating Br^- or I^- into bulk ice from liquid water is

about 1.6 eV or 1.4 eV, following the sequence of $F^- > Br^- > I^-$ in term of magnitude of energy requirement. This conclusion is qualitatively the same as that drawn from the MD simulations using the more accurate Nos é-Hoover 4-element chain thermostat.

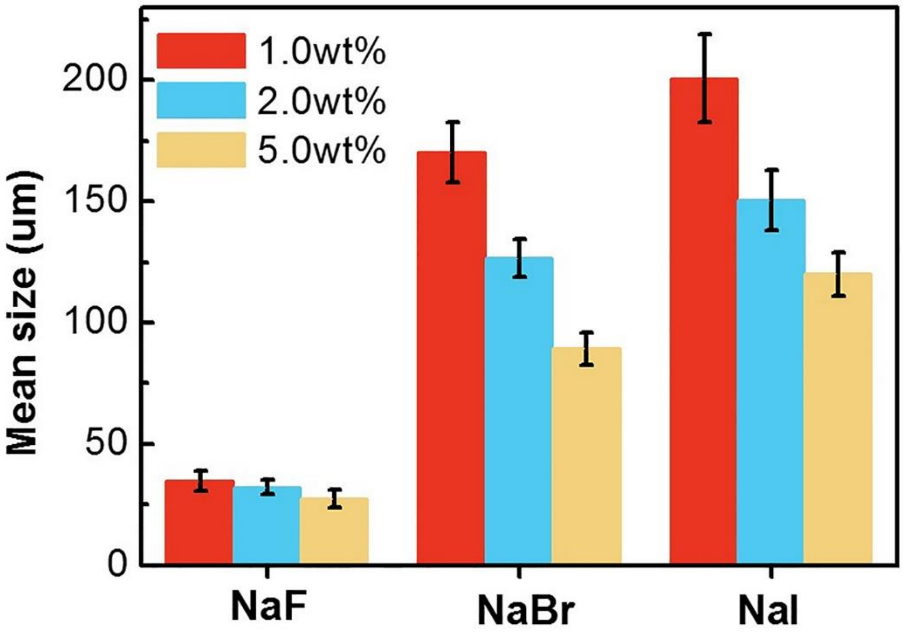

Supplementary Figure 9 | The average size of ice grains formed in 0.01 M salt solution within collagen after annealing at -6 °C for 45 min. With the same content of collagen, the Ion specificity in ice recrystallization remains. When the collagen concentration is 1.0 wt%, the average size of ice grains from NaF, BaBr and NaI solutions are 34.6±4.2 μm, 170.2±12.4 μm, 200.7±18. 1μm, respectively. For the same salt solution, the average size of ice grain decreases as the collagen concentration increases. For example, the average size of ice grains from 1.0 wt%, 2.0 wt% and 5.0 wt% collagen are 200.7±18.1 μm, 150.5±12.4 μm and 127.7±12.4 μm, respectively, when the concentration of NaI solution is 0.01 M.

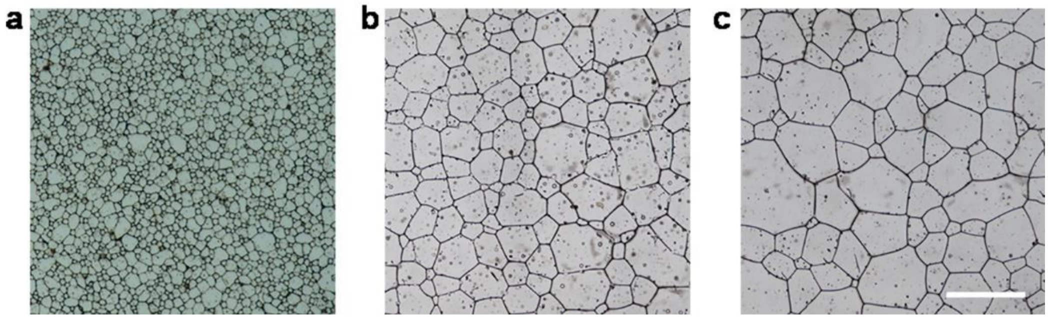

Supplementary Figure 10 | Optical images of meshes of NaF, NaBr and NaI after sublimation of ice grains by freezing drying. The concentration of the three salts solution is all 0.1 M. The formation of salt meshes indicates that most salts are located between ice grains after the annealing. Sea water contains 3.5 wt% dissolved salts (mainly sodium chloride). However, there is little salt in sea ice. These salt meshes give direct evidence of salt ejection by ice and explain the phenomenon of internal cleaning of sea ice[3, 4]. The white scale bar is 200 μm.

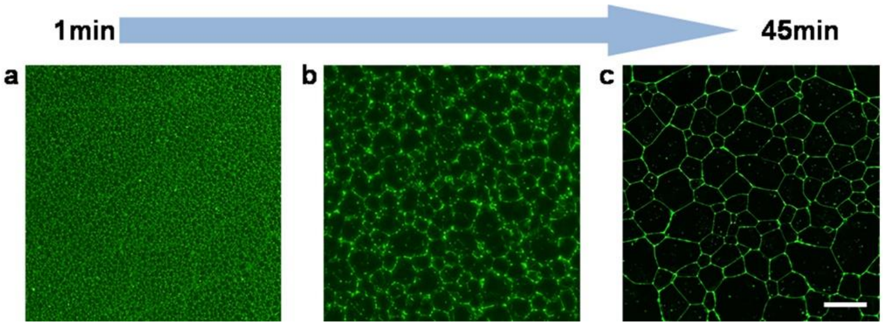

Supplementary Figure 11 | Fluorescence microscopy images of polycrystalline ice from 0.1 wt% quantum dot (CdSe capped by PEO) solution within 0.01 M NaI solution *versus* the annealing time. During the annealing process, the quantum dots are relocated, assembled between ice grains[5] and then form into meshes. The white scale bar is 200 μm.

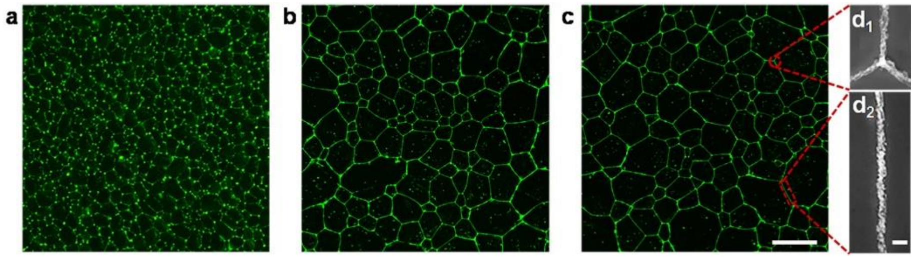

Supplementary Figure 12│Fluorescence microscopy images of quantum dot meshes. The mesh sizes can be tuned by putting in different NaF, NaBr and NaI solutions. a, b, and c show the quantum dot meshes within NaF, NaBr and NaI after sublimation of ice. d, SEM image of the joint and line. Scale bars for a, b, and c are 200 μm; scale bars of d_1 and d_2 are 500 nm.

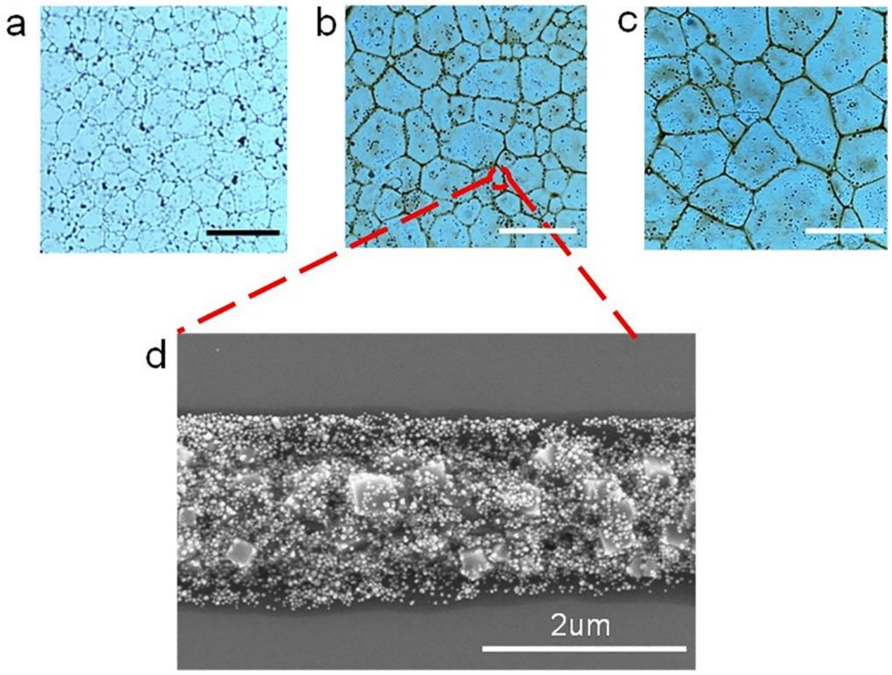

Supplementary Figure 13│Optical images of Ag nanoparticle meshes within NaF, NaBr and NaI. d shows the magnified line. Scale bars, a, 40 μm; b and c, 200 μm; d, 2 μm.

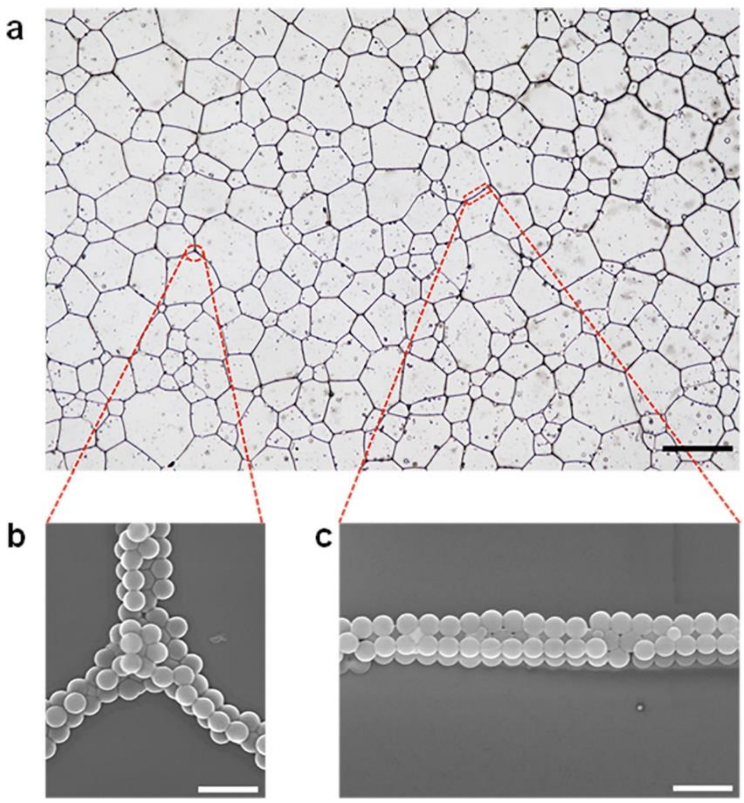

Supplementary Figure 14 │ Optical and SEM images of meshes composed polystyrene nanoparticle. b shows a joint, and c shows a line. The black/white sale bars: a, 200 μm; and b and c, 2 μm.

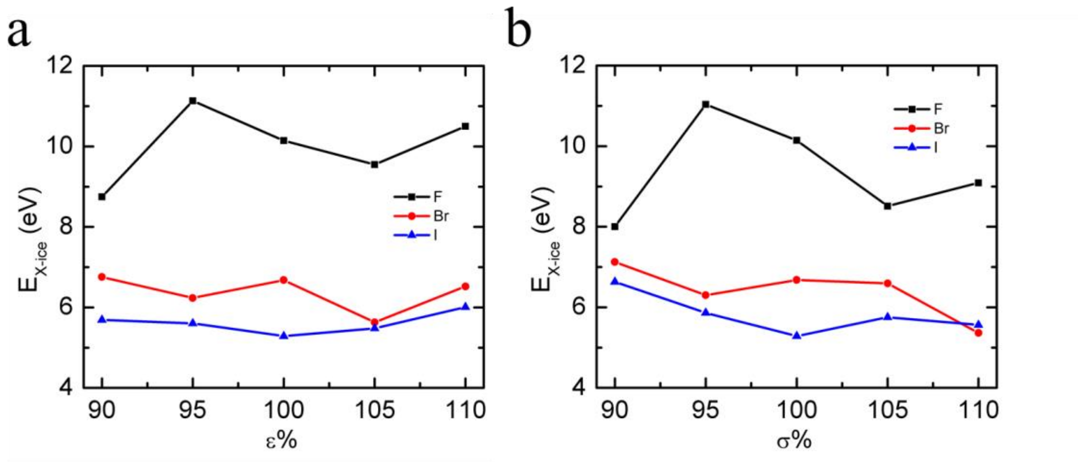

Supplementary Figure 15 │ Computed average interaction energy of an ion with surrounding water molecules in bulk ice *versus* the percentage change in ε (a) and the percentage change in σ (b) potential parameters. These test MD simulations show that our conclusion is reliable

and quite universal, insensitive to the changes in LJ potential parameters of the ions (at least within 10%).

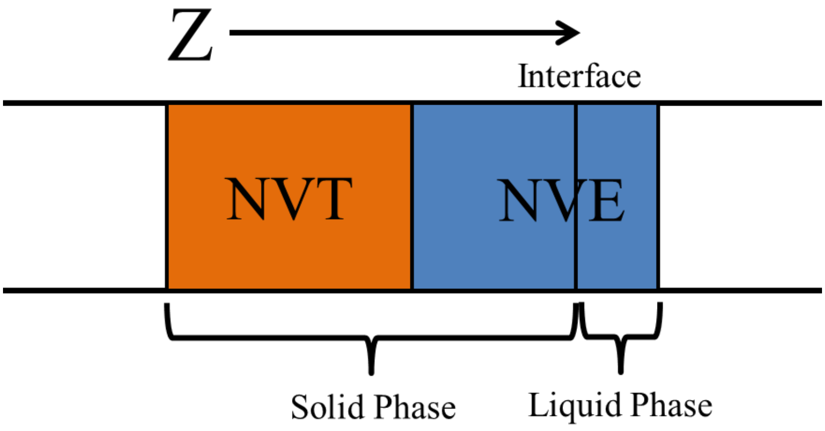

Supplementary Figure 16 | A hybrid microcanonical-canonical ensemble[6] is adopted in our MD simulations, in which the molecules whose centers of mass lie in a half region of the simulation box far from the interface are subjected to NVT dynamics, while the molecules lie in the other half region of the simulation box are subjected to NVE dynamics (include the solid/liquid interface).

Supplementary Table 1 | One way Anova analysis on the mean size of different anions (the same data from Fig. 2a).

concentration	Source of variation	DP	Sum of squares	Mean square	F	$F_{0.05}$	$F_{0.01}$	
0.1M	Model	9	1.16×10^6	128399.50	1025.64	1.91	2.47	$F>F_{0.01}$
	Error	290	3.63×10^4	125.19				
0.05M	Model	9	1.45×10^6	160639.08	935.74	1.91	2.47	$F>F_{0.01}$
	Error	290	4.98×10^4	171.67				
0.01M	Model	9	1.81×10^6	201606.67	830.85	1.91	2.47	$F>F_{0.01}$
	Error	290	7.04×10^4	242.65				
0.005M	Model	9	1.38×10^6	152945.20	800.97	1.91	2.47	$F>F_{0.01}$
	Error	290	5.54×10^4	190.95				
0.001M	Model	9	2.29×10^6	254004.03	688.69	1.91	2.47	$F>F_{0.01}$
	Error	290	1.07×10^4	368.82				

Supplementary Table 2 | One way Anova analysis on the mean size of different cations (the same data with Fig. 2b).

concentration	Source of variation	DP	Sum of squares	Mean square	F	$F_{0.05}$	$F_{0.01}$	
0.1M	Model	8	428759.89	53594.97	443.08	1.97	2.58	$F>F_{0.01}$
	Error	261	31569.51	120.96				
0.05M	Model	8	579542.20	72442.78	490.80	1.97	2.58	$F>F_{0.01}$
	Error	261	38522.76	147.60				
0.01M	Model	8	655030.79	81878.85	302.44	1.97	2.58	$F>F_{0.01}$
	Error	261	70659.95	270.73				
0.005M	Model	8	630124.87	78765.61	341.29	1.97	2.58	$F>F_{0.01}$
	Error	261	60235.97	230.79				
0.001M	Model	8	592702.67	74087.83	218.52	1.97	2.58	$F>F_{0.01}$
	Error	261	88488.23	339.04				

Supplementary Table 3│One way Anova analysis on the mean size of different quenching temperature (the same data with Fig. 3b).

	Source of variation	DP	Sum of squares	Mean square	F	$F_{0.05}$	$F_{0.01}$	
NaF	Model	5	34.88	6.98	0.29	2.27	3.12	$F<F_{0.05}$
	Error	174	4160.75	23.91				
NaBr	Model	5	829.21	165.84	0.83	2.27	3.12	$F<F_{0.05}$
	Error	174	34690.24	199.37				
H_2O	Model	5	366.60	73.32	0.47	2.27	3.12	$F<F_{0.05}$
	Error	174	27253.04	156.63				
NaI	Model	5	1675.39	335.08	0.77	2.27	3.12	$F<F_{0.05}$
	Error	174	76021.96	436.91				

Supplementary Table 4│One way Anova analysis on the mean size of different annealing temperature (the same data with Fig. 3c).

	Source of variation	DP	Sum of squares	Mean square	F	$F_{0.05}$	$F_{0.01}$	
NaF	Model	3	4162.49	1387.50	59.39	2.68	3.96	$F>F_{0.01}$
	Error	116	2710.20	23.36				
NaBr	Model	3	409767.14	136589.05	1773.88	2.68	3.96	$F>F_{0.01}$
	Error	116	8931.52	77.00				
H_2O	Model	3	304342.52	101447.51	1761.24	2.68	3.96	$F>F_{0.01}$
	Error	116	6681.44	57.60				
NaI	Model	3	679671.56	226557.19	1397.81	2.68	3.96	$F>F_{0.01}$
	Error	116	18800.81	162.08				

Supplementary Table 5│One way Anova analysis on the mean size of different concentration (the same data with Fig. 3d)

	Source of variation	DP	Sum of squares	Mean square	F	$F_{0.05}$	$F_{0.01}$	
NaF	Model	7	375688.33	53669.76	305.40	2.05	2.72	$F>F_{0.01}$
	Error	232	40770.88	175.74				
NaBr	Model	7	225919.34	32274.19	133.80	2.05	2.72	$F>F_{0.01}$
	Error	232	55961.67	241.21				
NaI	Model	7	454301.75	64900.25	128.22	2.05	2.72	$F>F_{0.01}$
	Error	232	117427.68	506.15				

Supplementary Table 6 | Two-factor factorial experiments (the data show the mean size of crystal size) unambiguously show that the ion species main effect is always the largest.

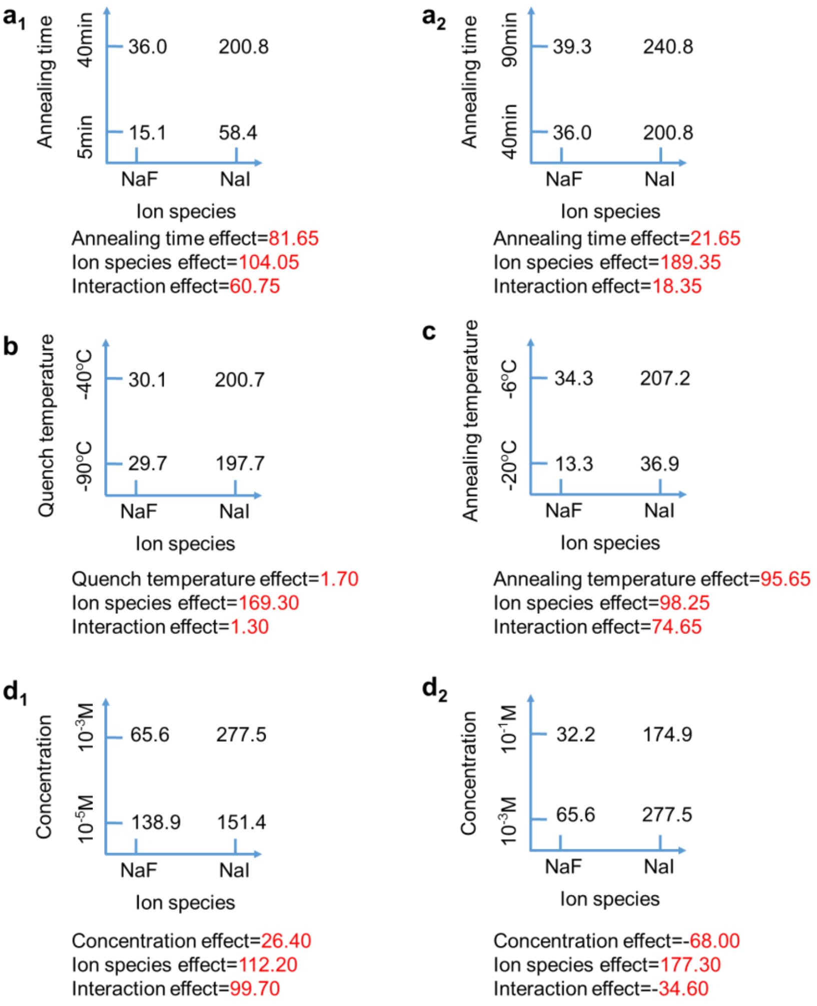

Supplementary References

1. Sun, Y., Xia, Y. Shape-Controlled Synthesis of Gold and Silver Nanoparticles. *Science* **298**, 2176-2179 (2002).

2. Ko, H. Y., Park, J., Shin, H., Moon, J. Rapid Self-Assembly of Monodisperse Colloidal Spheres in an Ink-Jet Printed Droplet. *Chem. Mater.* **16**, 4212-4215 (2004).

3. Menger, F. M., Galloway A. L., Chlebowski M. E., Apkarian RP. Ultrastructure in Frozen/Etched Saline Solutions: On the Internal Cleansing of Ice. *J. Am. Chem. Soc.* **126**, 5987-5989 (2004).

4. Cheng, J., Soetjipto, C., Hoffmann, M. R., Colussi, A. J. Confocal Fluorescence Microscopy of the Morphology and Composition of Interstitial Fluids in Freezing Electrolyte Solutions. *J. Phys. Chem. Lett.* **1**, 374-378 (2010).

5. Shen, X., Chen, L., Li, D., Zhu, L., Wang, H., Liu, C., *et al.* Assembly of colloidal nanoparticles directed by the microstructures of polycrystalline ice. *ACS nano* **5**, 8426-8433 (2011).

6. English, N. J. & MacElroy, J. M. Theoretical studies of the kinetics of methane hydrate crystallization in external electromagnetic fields. *J. Chem. Phys.* **120**, 10247-10256 (2004).
